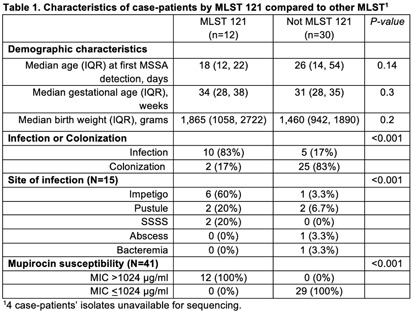# Investigation and control of an outbreak of methicillin-susceptible Staphylococcus aureus skin and soft tissue infections in a level IV NICU

**DOI:** 10.1017/ash.2024.133

**Published:** 2024-09-16

**Authors:** Gisel Rivera, Kara Mitchell, Jamie Marino, Vivien Yap, Liana Senaldi, Priyanka Tiwari, Jean-Marie Cannon, Rebecca Marrero Roldon, Marie-Claire Rowlinson, Wolfgang Haas, Rae-Jean Hemway, David Calfee, Lisa Saiman, Lars Westblade, Karen Acker

**Affiliations:** Weill Cornell Medicine- NYP; Wadsworth Center; Weill Cornell Medicine, New York, NY, USA; Weill Cornell; Florida Department of Health; NYSDOH; NewYork Presbyterian Hospital; Columbia University Irving Medical Center

## Abstract

**Background:** Neonatal intensive care units (NICU) outbreaks caused by methicillin-susceptible Staphylococcus aureus (MSSA) are less commonly reported than outbreaks caused by methicillin-resistant S. aureus. We report an unusual outbreak of MSSA skin and soft tissue infections (SSTIs) in a level IV NICU investigated by whole genome sequencing (WGS) and molecular typing. **Methods:** An investigation was initiated in a 56-single-bed NICU after four patients developed MSSA SSTIs in Week 1. Case-patients had positive MSSA cultures identified by clinical cultures or surveillance sampling (bilateral nares, axillae, umbilicus, and groin), and antibiotic susceptibility testing was performed. WGS and assessment of isolate relatedness through mutation event analysis and multi-locus sequence typing (MLST) was performed by the NYS DOH. Demographic and isolate characteristics were compared using Wilcoxon rank sum test, Fisher’s exact test and Pearson’s Chi-squared test, as appropriate. **Results:** From Week 2 to Week 32, 9 rounds of surveillance for MSSA colonization were conducted. In all, 30 case-patients had MSSA colonization and 16 infants developed infections including impetigo (n=7), pustules (n=5), staphylococcal scalded skin syndrome (SSSS, n=2), abscess (n=1), and bacteremia (n=1). All SSTI cases presented on infants’ faces, all of whom were on non-invasive respiratory support. MLST identified 4 distinct types including MLST 121 (n=12), MLST 398 (n=10), MLST 30 (n=6), and MLST 15 (n=6). Eight isolates were unrelated to other isolates. MLST 398 and MLST 30 included isolates not closely related (>9 mutation events). The 12 MLST 121 isolates were closely related (≤9 mutational events between all isolates), harbored the mupA gene, and were mupirocin-resistant (MIC>1024 ug/ml). Clinical infection and mupirocin resistance were associated with MLST 121 (Table 1). Multiple infection control measures were implemented, including increased availability of alcohol-based hand sanitizers, introducing bare-below-the-elbows practice for staff, contact precautions for case-patients, decolonization with mupirocin and chlorhexidine baths, environmental cleaning/disinfection, and removing excess equipment and supplies. No new cases of mupirocin-resistant or MLST 121 SSTIs occurred after Week 25. **Conclusion:** We report a MSSA outbreak associated with multiple MLST types and a predominant mupirocin-resistant strain. This report highlights the ability of molecular typing to characterize strains causing infections versus colonization and the potential loss of mupirocin as a control measure when outbreaks are caused by mupirocin-resistant strains. WGS analysis allows for increased discrimination of mutation events allowing for improved resolution of case relatedness compared to other typing methods. Successful control of this outbreak was achieved with a multitude of infection prevention and control.

**Figure:**
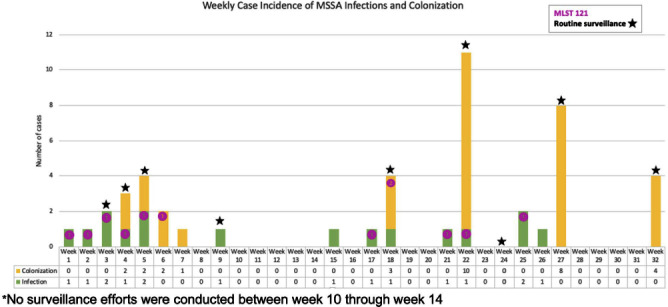

**Figure:**